# Unraveling the association between major depressive disorder and senescent biomarkers in immune cells of older adults: a single-cell phenotypic analysis

**DOI:** 10.3389/fragi.2024.1376086

**Published:** 2024-04-11

**Authors:** Erica C. Lorenzo, Jovany E. Figueroa, Derya A. Demirci, Ferris El-Tayyeb, Billy J. Huggins, Medha Illindala, Jenna M. Bartley, Laura Haynes, Breno S. Diniz

**Affiliations:** ^1^ UConn Health Center on Aging, University of Connecticut School of Medicine, Farmington, CT, United States; ^2^ Ponce Health Sciences University School of Medicine, Ponce, PR, United States

**Keywords:** senescence biomarkers, inflammation, aging, immune cells, major depressive disorder

## Abstract

**Background:** Little is known about the prevalence of cellular senescence among immune cells (i.e., immune cells expressing senescence markers, iSCs) nor is there a gold-standard to efficiently measure iSCs. Major depressive disorder (MDD) in older adults has been associated with many hallmarks of senescence in whole blood, leukocytes, and plasma, supporting a strong connection between iSCs and MDD. Here, we investigated the prevalence and phenotype of iSCs in older adults with MDD. Using a single-cell phenotypic approach, circulating immune cells were examined for iSC biomarkers and their relationship to depression and inflammation.

**Results:** PBMCs from older adults with MDD (aged 69.75 ± 5.23 years) and healthy controls (aged 71.25 ± 8.8 years) were examined for immune subset distribution and senescence biomarkers (i.e., lack of proliferation, senescence-associated heterochromatin foci (SAHF), and DNA damage). Dual-expression of SAHF and DNA damage was categorized by low, intermediate, and high expression. A significant increase in the number of high expressing total PBMCs (*p* = 0.01), monocytes (*p* = 0.008), a trending increase in the number of high expressing CD4 T cells (*p* = 0.06) was observed overall in those with MDD. There was also a significantly lower proportion of intermediate expressing cells in monocytes and CD4 T cells in MDD (*p* = 0.01 and *p* = 0.05, respectively). Correlation analysis revealed associations between iSCs and mRNA expression of factors related to SASP and immune cell function.

**Conclusion:** MDD is associated with increased senescent cell biomarkers in immune cell populations delineated by distinct levels of SAHF and DNA damage. Inflammatory markers might serve as potent indicators of iSC burden in MDD.

## Introduction

Cellular senescence is a state of irreversible replicative arrest initiated by different cell-cycle regulating pathways following extensive extracellular or intracellular stress. This process is critical for tissue homeostasis and prevention of tumorigenesis (Campisi, 2001; Reyes et al., 2022). However, senescent cells (SCs) accumulate with aging and have been implicated in the pathophysiology of many diseases and adverse health outcomes (Baker et al., 2011; Xu et al., 2018). Senescent cells have many important phenotypic features, such as the overexpression of p16^INK4a^ or p21^Cip1^, DNA damage, senescence-associated heterochromatin foci (SAHF), presence of senescence-associated β-galactosidase (SA-β-gal), shortened telomeres, mitochondrial damage, altered nutrient sensing and cell signaling, and morphological changes (González-Gualda et al., 2021). Another characteristic feature of cellular senescence is the manifestation of the senescence-associated secretory phenotype (SASP) consisting of many soluble and insoluble factors and proteins including inflammatory cytokines and chemokines, proteases, growth factors, and matrix proteins (Coppé et al., 2010). These features can differ greatly between cell types and have posed a challenge in establishing universal SC biomarkers. As a result, cell-specific markers have been more useful in SC identification.

Age-associated changes in immune function, often referred to as immunosenescence, are evident in the innate and adaptive immune systems. For example, immunosenescence of T cells is characterized by expression of surface markers of exhaustion [PD-1 (Janelle et al., 2021)], inhibition [CD57 (Brenchley et al., 2003) and KLRG-1 (Henson and Akbar, 2009)], decreased expression of co-stimulatory molecules [CD27 and CD28 (Plunkett et al., 2007)], CD45RA re-expression on memory cells (Henson et al., 2012), diminished CD45RA expression on naïve cells (Lambert et al., 2022), and p16 overexpression (Janelle et al., 2021). Many of these markers and functional changes, however, are integral to homeostatic mechanisms and physiological responses and are therefore not exclusively indicative of cellular senescence (Brenchley et al., 2003; Plunkett et al., 2007; Henson and Akbar, 2009; Henson et al., 2012; Janelle et al., 2021; Lambert et al., 2022). Infection models have also demonstrated that aged human T-cells may exhibit characteristics of senescence such as p21 gene expression, enrichment for SASP genes, and increased SA-β-gal following replicative stress but show repressed histone expression (Kim et al., 2021). Inhibition of SIRT1, a key regulator of genome stability, was shown to significantly augment histone expression, thereby enabling T-cells to re-enter the cell cycle, mitigate inflammation, and improve viral clearance (Kim et al., 2021). Classical senescence markers have also been observed in monocytes including the expression of SASP factors and elevated levels of inflammatory cytokines (Merino et al., 2011). Emerging evidence supports cell surface expression of CD36, a cell adhesion molecule and scavenger receptor, and p21 gene expression may serve as robust indicators of monocyte cellular senescence (Chong et al., 2018; Lin et al., 2023).

A definitive panel of markers that reliably identifies cellular senescence in immune cells, particularly concerning their incapacity to re-enter the cell cycle, however, remains to be established. Investigations into the relationship between senescence markers and disease, conducted primarily on bulk peripheral blood mononuclear cells (PBMCs) or total CD3-expressing cells, lack granularity regarding the contribution of immune cell subtypes (e.g., CD4 T-cells, CD8 T-cells, monocytes, etc.) expressing classical senescence markers to diverse pathological contexts. Taken altogether and given the broad and ambiguous nature of the generalized term “immunosenescence,” our preliminary study focuses specifically on immune cells exhibiting characteristics of cellular senescence described below, hereafter referred to as iSCs.

A growing body of evidence suggests a significant association between major depressive disorder (MDD) in older adults and increased cellular senescence characteristics (Lorenzo et al., 2023). Our previous studies have shown key inflammatory factors part of the SASP are strongly correlated with MDD, medical comorbidities, and severity of cognitive impairment (Diniz et al., 2019; Seitz-Holland et al., 2023). Our previous studies also suggest that higher SASP index scores are correlated with higher rates of non-remission of MDD following treatment (Diniz et al., 2022). MDD has also been associated with telomere attrition and DNA damage (Diniz et al., 2018; Gonçalves et al., 2021; Mendes-Silva et al., 2021; Vieira et al., 2021). Immune cells from adults with MDD exhibit elevated levels of oxidative damage alongside single and double stranded DNA breaks, coupled with a deficit in repair mechanisms, a common trait of SCs (Czarny et al., 2015; Kucuker et al., 2022). Additionally, heightened depression severity and prolonged duration of depressive symptoms correlate with shorter telomere length implying accelerated aging processes (Verhoeven et al., 2014).

Recent investigations utilizing a DNA methylation metric based on age-related epigenetic changes termed ‘GrimAge’ demonstrated that adults with MDD have a significantly higher GrimAge relative to their chronological age when compared to control non-depressed age-matched counterparts (Protsenko et al., 2021). Furthermore, another recent study showed the potential efficacy of DNA methylation risk scores as an effective predictor of MDD (Barbu et al., 2021). These findings collectively emphasize the intricate relationship between MDD and biomarkers of cellular senescence, particularly related to inflammation, DNA damage, and epigenetic alterations such as enhanced DNA methylation. These studies also highlight the need for a deeper exploration into the role of cellular senescence in the pathophysiology of MDD. To our knowledge, no prior study has evaluated the association between MDD and elevated iSC burden characterized by simultaneous expression of senescence biomarkers, nor if these cells contribute to the higher inflammatory markers observed in this condition.

In this study, we aimed to investigate differences in iSC burden in older adults with MDD when compared to non-depressed age-matched control individuals. To this end, we devised a strategy to identify iSCs at a single-cell resolution using imaging flow cytometry by combining markers of non-proliferation (Ki67^-^), double-stranded DNA breaks (γH2AX), and senescence-associated heterochromatin foci (SAHF, H3K9me2), an indicator of DNA methylation foci. This allowed for more careful and specific determination of iSC marker expression as opposed to traditional flow cytometry analysis. These markers were chosen given the previously noted variability in expression of conventional monocyte and T-cell surface markers and cell cycle regulators (e.g., p16, p21) with aging. Although double-stranded DNA breaks and DNA methylation foci are not exclusive features of cellular senescence on their own, evaluating dual expression of γH2AX and SAHF together may be a more reliable indicator of cellular senescence (Aird and Zhang, 2013; Paluvai et al., 2020). Therefore, we have used this method to distinguish iSCs within CD4, CD8, and monocyte cell populations. Additionally, we examined the association between iSCs and inflammatory changes commonly observed with aging and MDD episodes (Seitz-Holland et al., 2023).

## Materials and methods

### Sample and assessments

We included 32 older adults aged 60 years and older (16 with MDD and 16 never-depressed controls). All participants were screened for eligibility and recruited in the UConn Center on Aging, UConn Health, CT from November 2021 to December 2022. All participants also provided written informed consent to participate in the study. The diagnosis of a major depressive episode was made based on the DSM-5 diagnostic criteria (Diagnostic and statistical manual of mental disorders, 2013). All participants were not under antidepressant treatment at the time of psychiatric assessment and blood draw for flow cytometry analyses. We included 16 individuals with no prior or current history of major depressive episode or other major psychiatric diagnosis as a never-depressed control group. Additional exclusion criteria for this study were: 1) current diagnosis of bipolar disorder, schizophrenia, or other psychotic disorder; 2) history of intellectual disability, severe head trauma, or dementia; 3) alcohol or substance abuse or dependence (past 6 months) (except tobacco); 3) history of central nervous system disorder; 4) history or current evidence of stroke; 5) history of rheumatologic and other major inflammatory disorders; 6) current unstable medical illnesses; 7) history of infectious diseases in the month before recruitment assessment; 8) chronic use of anti-inflammatory medications (corticosteroids, AINES, immunomodulators).

All participants underwent a detailed structured psychiatric interview to confirm the diagnosis of a major depressive episode or confirm the absence of prior or current history of major depressive episode or other major psychiatric diagnosis. The severity of depressive symptoms was evaluated by the Montgomery-Asberg Depression Rating Scale (MADRS) (Montgomery and Asberg, 1979). Medical and psychiatric impairment was measured by the Cumulative Illness Rating Scale-Geriatric (CIRS-G). We used the Montreal Cognitive Assessment (MoCA) (Nasreddine et al., 2005) as a measure of global cognitive performance and to exclude possible dementia cases. Table 1 shows the baseline participant characteristics.

**TABLE 1:** Participant characteristics.

	MDD (*n* = 16)	Control (*n* = 16)	*p*-value
Sex			
Male, n (%)	10 (62.50)	5 (31.25)	0.100
Female, n (%)	6 (37.50)	11 (68.75)	
Age, Mean ± SD	69.75 ± 5.23	71.25 ± 8.80	0.740
Race	14 (87.50)	15 (93.75)	
White, n (%)	0 (0)	1 (6.25)	
Black or African American, n (%)	0 (0)	0 (0)	
Asian, n (%)	0 (0)	0 (0)	
American Indian/Alaska Native, n (%)	0 (0)	0 (0)	
Hawaiin or Pacific Islander, n (%)	0 (0)	0 (0)	
Unknown or not reported, n (%)	0 (0)	0 (0)	
More than one race, n (%)	2 (12.50)	0 (0)	
Other, n (%)	2 (12.50)	0 (0)	
Ethnicity	14 (87.50)	16 (100)	
Hispanic or Latino, n (%)	0 (0)	0 (0)	
Not Hispanic or Latino, n (%)			
Unknown or not reported, n (%)			
MADRS Total Score, Mean ± SD	21.81 ± 5.26	0.75 ± 1.4	0.0001
CIRS-G Total Score, Mean ± SD	11.75 ± 3.15	5.79 ± 3.79	0.0001
Total PBMC Count, Mean	3.2 × 10^7^ ± 1.7 × 10^7^	2.8 × 10^7^ ± 1.5 × 10^7^	0.5200

BMI, body mass index; CIRS-G, Cumulative Illness Rating Scale-Geriatric; MDD, major depressive disorder; MADRS, Montgomery–Asberg Depression Rating Scale; PBMC, peripheral blood mononuclear cells.

### Blood collection and processing

Non-fasting whole blood was collected using EDTA tubes totaling 30 mL of blood (BD Vacutainer, K2 EDTA 10.8 mg, Ref #: 367863), between 9 and 11 a.m. and immediately processed following a standardized protocol described below. Fresh blood was used to isolate PMBCs and evaluate iSCs.

### PBMC isolation

Following plasma separation by centrifugation and isolation, the blood tubes were gently inverted several times to re-mix the sample and diluted 1:1 with 1x PBS. The samples were layered over Ficoll-Paque PLUS (Cytiva) forming a density gradient upon centrifugation (400 × g for 40 min, 7 acceleration, 0 deceleration) from which we isolated the PBMCs. Any remaining red blood cells were lysed using RBC Lysis Buffer (Thermo Scientific), and the total PBMC count (from 30 mL of blood) was determined using the Cellometer cell counter following resuspension in 10 mL PBS. Aliquots of 5 × 10^6^ cells were used for PBMC subtyping and the remaining PBMCs were cryopreserved and stored for future investigations.

### PBMC and iSC phenotyping

Fresh PBMC aliquots were incubated with Human TruStain FcX™ (BioLegend) to inhibit non-specific binding of antibodies and NucBlue™ Live Cell Stain ReadyProbes™ (Hoechst 33342, invitrogen) to label cellular DNA. Cells were then incubated with surface-binding antibodies specified in Supplementary Table S1 to mark CD4 and CD8 T cells, B cells (for exclusion), NK cells (for exclusion), and monocytes. After washing steps and fixation with 2% paraformaldehyde, cells were permeabilized using 0.1% Triton X-100 (Sigma) in PBS and stained with intranuclear antibodies for senescence biomarkers (Supplementary Table S1). We used non-permeabilized PBMCs as a technical control to confirm the antibody specificity and nuclear staining for the analysis.

After additional washing steps, the PBMCs were run on the AMNIS IMAGESTREAM^®X^ MARK II (Luminex Corp., TX) for single-cell identification of PBMC subtypes and senescence markers. The AMNIS IMAGESTREAM^®X^ MARK II combines both flow cytometry and microscopy functionalities, allowing the measurement of marker expression from a bulk cell population, while also allowing the capture of bright field and fluorescence images of each individual cell, thus providing information not only for the bulk of each cell population of interest, but also providing immunohistochemistry information at a single cell resolution. The general gating strategy is shown in Supplementary Figure S2A. After determining which cells were in focus, alive, and single-cells, lymphocytes were gated on and further evaluated for surface markers to distinguish different immune cell types. Because each cell type was determined from the total lymphocytes in each sample, all counts and percentages of immune cells were normalized to total PBMCs.

**FIGURE 1:**
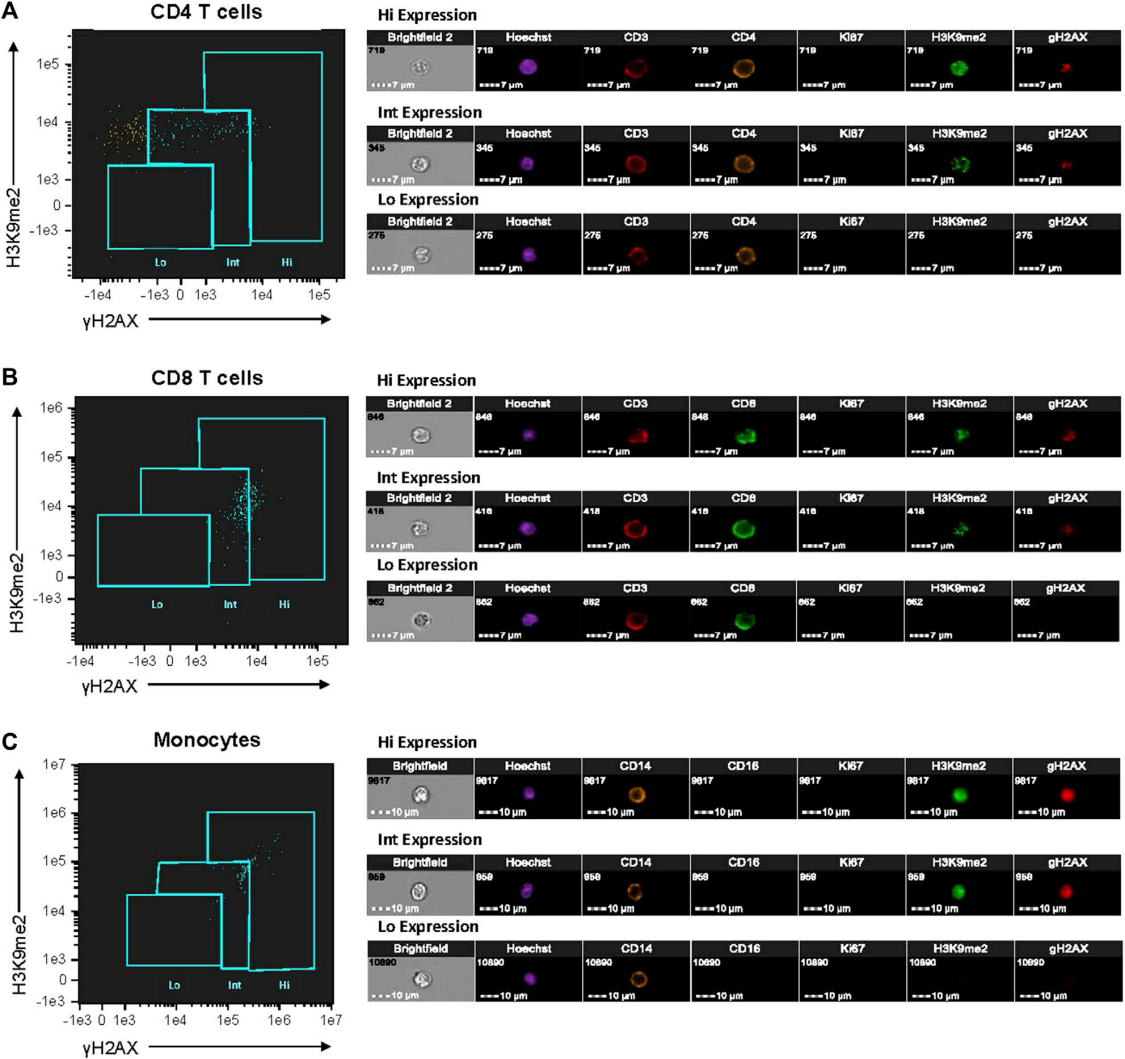
Characterization of cellular senescent biomarker expression in PBMCs from older adults with MDD. PBMCs isolated from fresh blood of study participants were fluorescently labeled with antibodies listed in Supplementary Table S1. Single-cell imaging flow cytometry was performed using Amnis instrumentation to assess dual-expression of SAHF and dsbDNA markers (H3K9me2 and γH2AX, respectively) in non-proliferative (Ki67^‒^) immune cells (CD4 T-cells, CD8 T-cells, and monocytes). Expression of senescence-associated markers were categorized by low (Lo), intermediate (Int), and high (Hi) levels. Single cell images of CD4 T-cells **(A)**, CD8 T-cells **(B),** and monocytes **(C)** from one individual with MDD are shown. All images were taken at 40× magnification. This analysis was performed on each study participant (MDD, *n* = 16; Control, *n* = 16). Images and cytometry data from acquisition were analyzed on IDEAS6.2 software. dsbDNA, DNA double-stranded breaks (as indicated by γH2AX labeled DNA damage foci); MDD, major depressive disorder; PBMC, peripheral blood mononuclear cell; SAHF, senescence-associated heterochromatin foci (as indicated by H3K9me2 labeled foci).

To evaluate senescence biomarkers, we first used Ki67 negativity (Ki67^-^) to a identify non-proliferative state within each immune cell subtype. Then, intranuclear senescence-associated antibody expression was used to identify senescence-associated heterochromatin foci (SAHF) by H3K9me2 and double-stranded DNA breaks (dsbDNA) by γH2AX. Based on the dual-expression patterns of both dsbDNA and SAHF within each non-proliferative (Ki67^-^) cell, we defined 3 distinct iSC phenotypes: low (Ki67^-^ SAHF^lo^ dsbDNA^lo^); intermediate (Ki67^-^ SAHF^int^ dsbDNA^int^); and high (Ki67^-^ SAHF^hi^ dsbDNA^hi^) (Supplementary Figure S2B **)**. Supplementary Figure S2 shows the general gating strategy and analysis of these populations based on the SAHF and dsbDNA dual-expression. In Figure 1, we demonstrate the phenotypic distribution of iSC biomarkers within CD4, CD8, and monocyte cell populations in older adults with MDD, characterizing diverse expression patterns of these markers. Cells showing the high senescence phenotype demonstrated a distinct phenotype characterized by prominent nuclear punctate areas of senescence markers, while those with intermediate senescence phenotype exhibited lower intensity and fewer punctate spots. Cells with low senescent phenotype showed minimal to no expression of senescence biomarkers or punctate spots (Figure 1). These cells could be non-senescent and maintain their ability to re-enter the cell cycle and/or could also be at the very early stages of DNA damage accumulation. All analyses for the PBMC count, identification of cell subtypes, and identification of iSCs were performed using IDEAS^®^ and FlowJo^®^ software.

### RNA isolation and cDNA synthesis

5 × 10^6^ fresh PBMCs were dissociated using TRIzol™ Reagent (invitrogen). RNA was isolated according to manufacturer protocol. RNA quantity and quality were assessed via Nanodrop 2000c (Thermo Scientific). RNA was reverse transcribed using iScript Advanced cDNA synthesis Kit (Bio-Rad Laboratories).

### RT-PCR

Real time-PCR was performed using predesigned commercially available primers (Bio-Rad) to determine mRNA expression levels of *IL-1β, IL-2, IL-5, IL-6, IL-8, IL-10, IL-13, IL-17α, IFN-γ, TNF-α, CCL-2,* and *CXCL-1*. A list of all assays and their unique assay ID are listed in Supplementary Table S2. 2.5 ng of cDNA was used for each cytokine PCR assay. Each assay was run in triplicate for each participant and was normalized to actin. Cq values were averaged together for each cytokine and control per participant to obtain an average Cq value. The ΔCq value was calculated by subtracting the average actin Cq value from the average cytokine Cq value. The ΔΔCq value was then calculated by subtracting the average of all control ΔCq values from the ΔCq value for each cytokine and control participant. Fold change was then determined by calculating 2^−ΔΔCq^.

### Statistical analysis

Descriptive analysis was performed to determine the distribution patterns of the clinical and biomarker data. We focused on the percentage and number of cells expressing negative, intermediate, and high senescence biomarkers for various PBMC subtypes (CD4 T cells, CD8 T cells, and monocytes). The data distribution from control and depressed individuals was skewed to the left with a high frequency of zero senescent cell counts (e.g., non-depressed control individuals often had no cells with high expression of senescence biomarkers). Since the use of parametric (e.g., *t*-test) or non-parametric tests (e.g., Mann-Whitney test) can be biased to this data distribution pattern, we opted to carry out Tobit regression models, with left censoring, to evaluate differences in the percentage of senescence cells phenotypes, and negative binomial regression or zero-inflated negative binomial regression to evaluate differences in the cell count of senescence cells phenotypes between older adults with major depression and non-depressed controls. Correlation analysis was performed using Spearman’s rho and Student’s t-test was performed where specified to compare MDD and control groups for various measures. Given the limited sample and exploratory nature of these analyses, we did not correct the *p*-value for multiple comparisons. All statistical analyses were done using STATA version 17 for Windows (College Station, TX), IBM SPSS Statistics version 29 for Windows, R software version 4.3.2 for Windows, or GraphPad Prism 9 version 9.3.1 for Windows.

## Results

### Participant characteristics

In this preliminary study we included 32 older adults aged 60 years and older (16 with MDD, mean age 69.75 ± 5.23 years and 16 never-depressed controls, mean age 71.25 ± 8.80 years). Other inclusion and exclusion criteria as well as psychological assessments used to evaluate depressive symptoms and cognition are as described in the Methods. Demographic information, assessment scores evaluating depression severity (Montgomery-Asberg Depression Rating Scale, MADRS) and medical and psychiatric impairment (Cumulative Illness Rating Scale-Geriatric, CIRS-G), in addition to total peripheral blood mononuclear cell (PBMC) counts are reported in Table 1. There were no significant differences in age or sex distribution between MDD and control groups. As expected, individuals with MDD had higher MADRS scores and greater medical comorbidity burden, measured by the CIRS-G.

Current meta-analysis supports that older adults with depression exhibit dysregulation in the immune cell compartment compared to non-depressed individuals (Foley et al., 2023). Specifically, of the studies included in the analysis of each subtype, there was a marked increase in the mean absolute counts of white blood cells, granulocytes, neutrophils, monocytes, CD4 T cells, natural killer cells, B cells, and activated T-cells in depression, compared to controls (Foley et al., 2023). It is, of course, possible that depression as a systemic disorder in itself, comorbidities associated with depression, the use of antidepressant medications, and many other genetic or lifestyle characteristics have an impact on immune cell counts and function. We, therefore, wanted to ensure our data was not confounded by skewed overall immune cell counts to be able to compare MDD and control individuals. Along with our exclusion criteria described in the Methods, study participants were not undergoing antidepressant treatment. When evaluating cell counts and percentages, we found no significant difference in the total PBMC, CD4 T-cell, CD8 T-cell, or monocyte populations between MDD and control groups (Supplementary Figure S1 and Supplementary Table S3). All cell counts for each immune cell subset population (CD4, CD8, and monocyte) were normalized to the total PBMC number for each participant.

### Immune cell expression of cellular senescence markers (iSCs) in MDD

As described earlier, we chose a specific panel of markers to evaluate DNA damage foci, SAHF, and the lack of proliferation associated with senescence. Although further studies would be needed to ensure cells could not re-enter the cell cycle when exposed to stimulus, we have included this combination of markers that reliably characterizes the reinforcement of the cellular senescence phenotype when combined within each cell (Mah et al., 2010; Parris et al., 2015; Paluvai et al., 2020). In this study, immune cells that are not proliferative (Ki67^-^), have double-stranded DNA breaks (dsbDNA), and SAHF are considered to have markers of cellular senescence and will be referred to hereon as iSCs. We evaluated the total PBMC population and the CD4 T-cell, CD8 T-cell, and monocyte sub-populations. Given that traditional flow cytometry would only allow for bulk analysis, we used Amnis Imagestream (Amnis), providing us with a fine-grained view of iSCs. Amnis has several advantages, including capturing both bulk and single-cell phenotypic data. Because the overall population of senescent cells has been estimated to be quantifiably small, we wanted to ensure that signal from iSCs would not be overpowered by the substantial number of non-senescent cells that would have been observed in bulk analysis.

By utilizing the microscopy functions of Amnis analysis software, we could confirm the presence of both dsbDNA and SAHF within the same Ki67^-^ cell. We could also effectively select cells that had true staining within the nucleus, as opposed to those that were auto-fluorescing or had artifacts of antibody staining processes that would give false positive results, which is common in traditional flow cytometry bulk analysis. We also found that with the overall population of iSCs being very small, it would have been extremely difficult, if not nearly impossible, to distinguish cells from one another with varying levels of iSC markers with traditional flow. In Figure 1, we demonstrate the phenotypic distribution of iSC biomarkers within CD4, CD8, and monocyte populations in older adults with MDD, characterizing these diverse expression patterns. Although the differences seem not particularly prominent to the naked eye when looking at a flow plot, we found by the microscopy analysis that, in fact, there were Ki67^-^ cells that had high levels of both dsbDNA and SAHF (Hi), while those in the middle of the plots typically had much dimmer dsbDNA and SAHF (Int). We also were able to detect Ki67^-^ cells that had very low to no dsbDNA and SAHF (Lo). Even further, iSCs with Hi phenotype had numerous prominent nuclear punctate areas of senescence markers, while those with Int phenotype exhibited lower intensity and fewer punctate spots. Cells with Lo phenotype showed minimal to no senescence biomarkers or punctate spots (Figure 1).

**FIGURE 2:**
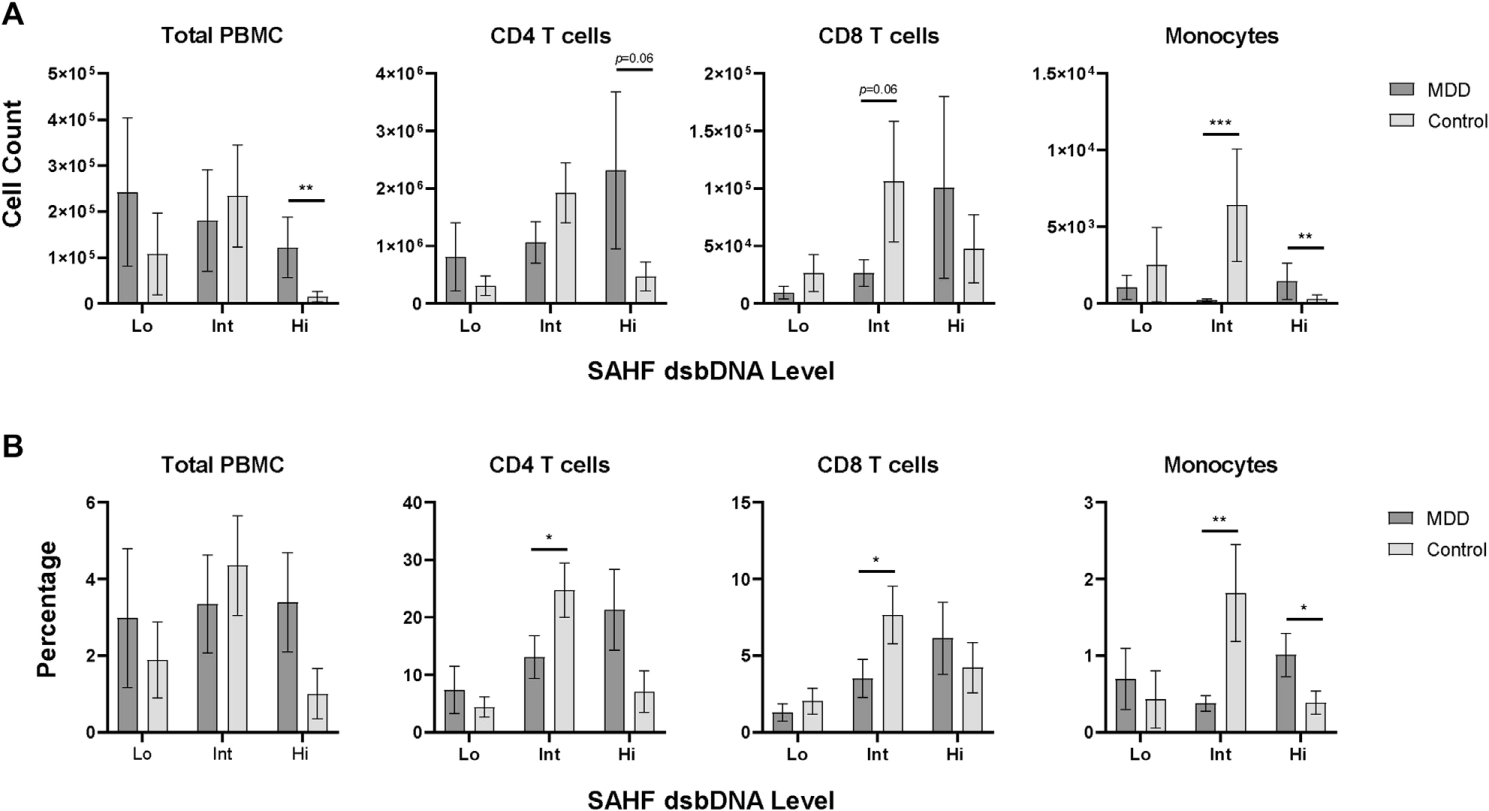
Senescence marker expression levels in PBMC subsets. PBMCs assessed for low (Lo), intermediate (Int), and high (Hi) expression of senescence-associated markers SAHF and dsbDNA, as shown in Figure 1, were quantified for each study participant (MDD, *n* = 16; Control, *n* = 16). Count **(A)** and percentage **(B)** of SAHF and dsbDNA levels are shown for the total PBMC population and each subpopulation in MDD and control groups (dark gray and light gray bars, respectively). Data was analyzed in GraphPad Prism software using Student’s T test to compare MDD and control groups. Bars represent mean SAHF dsbDNA expression levels for each population. Error bars represent standard error of the mean. *p* < 0.05. dsbDNA, DNA double-stranded breaks (as indicated by γH2AX labeled DNA damage foci); MDD, major depressive disorder; PBMC, peripheral blood mononuclear cell; SAHF, senescence-associated heterochromatin foci (as indicated by H3K9me2 labeled foci); SEM, standard error of the mean.

Although we will need to determine if these distinct phenotypes are relevant in cell function or differential contribution to MDD pathophysiology, we noticed this pattern was consistent within every older adult included in this study. In light of this finding, we compared this pattern of iSC Hi, Int, and Lo phenotypes between MDD and control individuals. Interestingly, those with MDD showed a significant elevation in the number of iSC Hi cells within the total PBMC population compared to controls (*p* = 0.01) (Figure 2A; Table 2). Monocytes also showed a significantly higher count of iSC Hi cells (*p* = 0.008) and a marked decrease in the number of iSC Int cells (*p* < 0.001) in individuals with MDD. We also identified a trend toward increased iSC Hi CD4 T cells (*p* = 0.06) and decreased iSC Int CD8 T cells (*p* = 0.06) in MDD.

**TABLE 2:** iSC phenotype in total PBMCs, CD4 and CD8 T cells, and monocytes.

		MDD	Control	Statistics
Total PBMC (Count)	Lo^a^	2.42 × 10^5^ ± 6.24 × 10^5^	1.07 × 10^5^ ± 3.57 × 10^5^	*β* = 0.81, z = 0.80, *p* = 0.42
	Int^a^	1.80 × 10^5^ ± 4.27 × 10^5^	2.34 × 10^5^ ± 4.44 × 10^5^	*β* = −0.30, z = −0.42, *p* = 0.67
	Hi^b^	1.22 × 10^5^ ± 2.55 × 10^5^	1.45 × 10^4^ ± 4.46 × 10^4^	*β* = 2.23, z = 2.49, *p* = 0.01
CD4 (Count)	Lo^b^	8.15 × 10^5^ ± 2.37 × 10^6^	3.10 × 10^5^ ± 6.71 × 10^5^	*β* = 1.25, z = 1.68, *p* = 0.09
	Int^b^	1.06 × 10^6^ ± 1.34 × 10^6^	1.92 × 10^6^ ± 2.09 × 10^6^	*β* = −0.52, z = −1.15, *p* = 0.25
	Hi^b^	2.31 × 10^6^ ± 5.28 × 10^6^	4.72 × 10^5^ ± 1.00 × 10^6^	*β* = 1.41, z = 1.85, *p* = 0.06
CD8 (Count)	Lo^b^	9304.32 ± 2.24 × 10^4^	2.64 × 10^4^ ± 6.44 × 10^c^	*β* = −0.86, z = −1.05, *p* = 0.29
	Int^b^	2.64 × 10^4^ ± 4.62 × 10^5^	1.06 × 10^5^ ± 2.09 × 10^4^	*β* = −1.24, z = −1.88, *p* = 0.06
	Hi^b^	1.00 × 10^5^ ± 3.06 × 10^5^	4.77 × 10^4^ ± 1.19 × 10^4^	*β* = 0.69, z = 0.75, *p* = 0.45
Monocyte (Count)	Lo^b^	1043.74 ± 3193.66	2531.64 ± 9756.03	*β* = −2.17, z = −1.55, *p* = 0.12
	Int^b^	206.28 ± 309.59	6414.96 ± 1.46 × 10^4^	*β* = −3.62, z = −4.44, *p* < 0.001
	Hi^b^	1452.71 ± 4762.04	284.12 ± 1058.89	*β* = 1.69, z = 2.66, *p* = 0.008
Total PBMC (%)	Lo^c^	2.98 ± 7.00	1.89 ± 3.96	*β* = −0.14, t = −0.06, *p* = 0.95
	Int^c^	3.35 ± 4.98	4.35 ± 5.21	*β* = −1.43, t = −0.76, *p* = 0.45
	Hi^c^	3.39 ± 5.02	1.01 ± 2.60	*β* = 2.66, t = 1.68, *p* = 0.10
CD4 (%)	Lo^c^	0.07 ± 0.18	0.06 ± 0.09	*β* = 1.43, t = 0.24, *p* = 0.80
	Int^c^	0.10 ± 0.13	0.25 ± 0.26	*β* = −12.29, t = −2.51, *p* = 0.05
	Hi^c^	0.44 ± 0.53	0.21 ± 0.47	*β* = 19.94, t = 1.55, *p* = 0.13
CD8 (%)	Lo^c^	0.01 ± 0.02	0.16 ± 0.44	*β* = −1.12, t = −0.77, *p* = 0.44
	Int^c^	0.26 ± 0.78	0.23 ± 0.20	*β* = −4.68, t = −2.01, *p* = 0.05
	Hi^c^	1.11 ± 2.23	0.49 ± 1.14	*β* = 2.71, t = .054, *p* = 0.59
Monocyte (%)	Lo^c^	0.69 ± 1.60	0.43 ± 1.49	*β* = −0.45, t = −0.58, *p* = 0.56
	Int^c^	0.38 ± 0.41	1.82 ± 1.82	*β* = −1.72, t = −2.56, *p* = 0.01
	Hi^c^	1.01 ± 1.13	0.39 ± 0.60	*β* = 0.77, t = 1.97, *p* = 0.05

^a^ Negative binomial regression.

^b^ Zero-inflated negative binomial regression.

^c^ Tobit regression. Lo, Int, Hi refer, respectively, to low, intermediate, and high expression levels of iSC markers identified by dual-expression of SAHF and dsbDNA. MDD was used as the reference group. Values are shown as mean ± standard deviation. *p* < 0.05. MDD, major depressive disorder; PBMC, peripheral blood mononuclear cells.

These differences in iSC counts were also reflected in the percentage of these phenotypes (Figure 2B; Table 2). Individuals with MDD demonstrated a significant increase in the percentage of iSC Hi monocytes (*p* = 0.05) and a significant reduction in the percentage of iSC Int monocytes (*p* = 0.01). Older adults with MDD also exhibited a significantly lower percentage of iSC Int CD4 and CD8 T cells (*p* = 0.05 for both). When iSCs across Hi, Int, and Lo phenotypes were analyzed collectively, however, the proportion of overall iSCs is quite low and not significantly different between MDD and control groups (*p* = 0.918). The mean percentage of iSCs in the total PBMC population (inclusive of Hi, Int, and Lo phenotype) was 3.24% (SD = 5.62) in the MDD group and 2.42% (SD = 4.23) in the control group.

### iSCs and inflammatory cytokine expression

**FIGURE 3:**
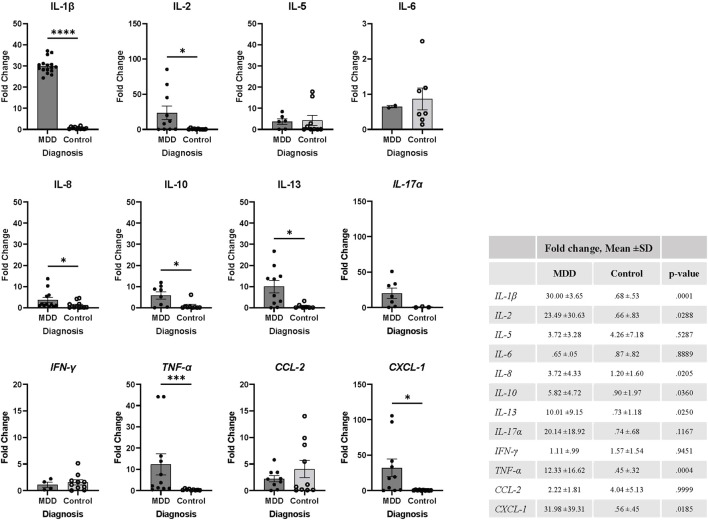
MDD is associated with elevation of inflammatory markers in PBMCs. mRNA expression levels were determined using fresh isolated PBMCs by RT-PCR from each study participant (MDD, *n* = 16; Control, *n* = 16). Values were normalized to actin expression levels and fold change was calculated using Cq values relative to the mean of control group cytokine mRNA expression, as described in methods. Expression for each cytokine was not detected in every participant and therefore excluded from the analysis. Data was analyzed in GraphPad Prism software using Student’s T test to compare MDD and control groups. Bars represent mean fold change for each population. Error bars represent standard error of the mean. The table includes mean ±SD and p-values, *p* < 0.05. MDD, major depressive disorder; SD, standard deviation.

As described earlier, our previous studies have demonstrated that SASP is strongly correlated with MDD severity, medical comorbidities, cognitive impairment, and higher rates of non-remission following anti-depressant treatment (Diniz et al., 2019; Diniz et al., 2022; Lorenzo et al., 2023; Seitz-Holland et al., 2023). While SASP levels have been notably elevated in MDD, their quantification has conventionally relied upon measurement in plasma. Despite the potential of plasma SASP levels as reliable indicators of MDD pathophysiology, discerning the source of SASP factors remains challenging. Additionally, many SASP factors are secreted by a myriad of different cell types, hindering our ability to determine whether they come from circulating cells or tissue resident cells and their magnitude of SASP factor production. Although *in vitro* cellular stimulation offers insight into SASP production dynamics within the immune cells of individuals with MDD, it does not allow us to evaluate SASP production signals at a basal level. For this reason, we evaluated mRNA expression levels of key cytokines that are associated with immune cells and known to be dysregulated with age including *IL-1β, IL-2, IL-5, IL-6, IL-8, IL-10, IL-13, IL-17α, IFN-γ, TNF-α, CCL-2, and CXCL-1* in total PBMCs of our study participants. We found that older adults with MDD have significantly elevated mRNA expression of *IL-1β* (*p* = 0.001), *IL-2* (*p* = 0.029), *IL-8* (*p* = 0.020), *IL-10* (*p* = 0.036), *IL-13* (*p* = 0.025), *TNF- α* (*p* = 0.0004), and *CXCL-1* (*p* = .018) when compared to controls (Figure 3).

**FIGURE 4:**
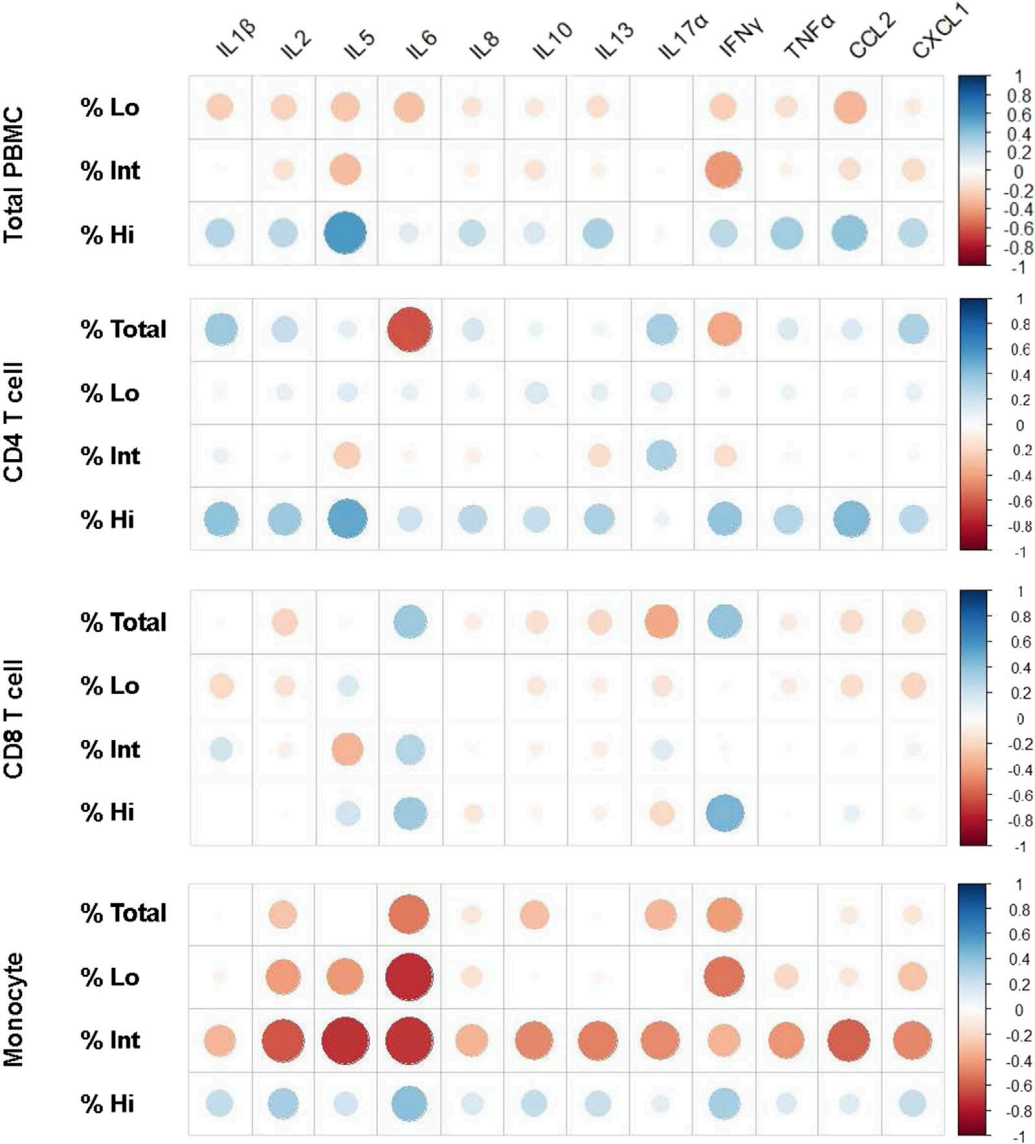
Spearman’s correlation graphs representing fold change of cytokine expression and percentage of iSC phenotypes in PBMCs. Spearman’s correlations were performed to determine the association between inflammatory cytokine markers, described in Figure 3 and iSC Hi, Int, and Lo phenotypes on the total PBMC population, CD4 T-cell, CD8 T-cell, and monocyte compartments. Data for each graph is included in Supplementary Table S4 and correlation graphs were generated using R studio software. Both MDD and control groups were included together in this analysis to determine the statistical trends in the overall dataset. Blue indicates an association in a positive direction whereas red indicates an association in a negative direction. dsbDNA, DNA double-stranded breaks (as indicated by γH2AX labeled DNA damage foci); iSC, immune cell expressing senescence biomarkers (Ki67^-^, dsbDNA^+^, and SAHF^+^); MDD, major depressive disorder; PBMC, peripheral blood mononuclear cell; SAHF, senescence-associated heterochromatin foci (as indicated by H3K9me2 labeled foci).

We next wanted to gain some initial insight as to whether immune cell mRNA expression of these cytokines has any relationship to the various iSC phenotypes within the whole study group (MDD and control individuals together). Spearman’s correlations revealed a significant positive association between IL-5 mRNA expression and the iSC Hi CD4 T cells (Figure 4 and Supplementary Table S4). Interestingly, we found a moderate to strong negative correlation between the iSC Int monocytes and *IL-2* (*p* = 0.002), *IL-5* (*p* = 0.003), *IL-6* (*p* = 0.024), *IL-10* (*p* = 0.026), *IL-13* (*p* = 0.026), *TNF-α* (*p* = 0.038), *CCL-2* (*p* = 0.007), and *CXCL-1* (*p* = 0.021) mRNA expression (Figure 4; Supplementary Table S4). It is worth noting that we found other moderate to strong associations (e.g., Rho> |0.4|) for several cytokines, but such associations were not statistically significant, possibly due to the limited sample size included in the analyses. Given these interesting findings, we conducted additional secondary correlation analyses between iSC phenotypes and cytokine mRNA expression within the MDD and control groups separately (Supplementary Table S5).

In the MDD group, a strong positive correlation was observed between iSC Hi CD4 T cells and mRNA expression of *IL-5* (Rho = 0.94, *p* = 0.015), *IL-8* (Rho = 0.65, *p* = 0.033), *IL-13* (Rho = 0.70, *p* = 0.019), and *CCL-2* (Rho = 0.80, *p* = 0.001). iSC Hi monocytes exhibited a very robust, positive correlation with *IL-5* mRNA expression (Rho = 0.96, *p* = 0.003). iSC Int or Lo CD8 T cells showed a strong and significant negative correlation with *IL-5* (Rho = −.83; *p* = 0.048), *IL-8* (Rho = −.64; *p* = 0.038), *IL-13* (Rho = −.73; *p* = 0.014), *IL-17α* (Rho = −.81; *p* = 0.021), *CCL-2* (Rho = −.76; *p* = 0.015), and *TNF-α* (Rho = −.66; *p* = 0.029) mRNA expression (Supplementary Table S5). A similar pattern emerged for the association between iSC Int or Lo monocytes and *IL-2* (Rho = −.66; *p* = 0.023) and *IL-5* (Rho = −.79; *p* = 0.039) mRNA expression. Comparable associations were observed within the control group, wherein iSC Int and Lo monocytes displayed a strong negative correlation with *IL-2* (Int, Rho = −.59; *p* = .046; Lo, Rho = −.58; *p* = 0.041), *IL-5* (Rho = −.68; *p* = 0.045), *IL-6* (Rho = −.85; *p* = 0.012), and *CCL-2* (Rho = −.75; *p* = 0.023) mRNA expression.

## Discussion

Our study provides valuable insights into the intersection between MDD in older adults and abnormalities in hallmarks of biological aging, focusing specifically on cellular senescence biomarkers in immune cells. Cellular senescence manifests differently across cell types, creating the need for cell-specific markers. This poses a challenge in evaluating multiple immune subsets simultaneously, as is often done, because of the abundance of cell surface and intracellular markers, senescence markers, and the substantial number of cells required for traditional cytometric evaluations, limiting the feasibility of large-scale analysis. Additionally, many of the markers currently used to identify aged “immunosenescent” cells are not exclusive markers of cellular senescence, but rather of general immune cell aging and exhaustion. While established markers such as SA-β-gal, p16^INK4a^, p21^Cip1^ have been evaluated in the senescence of various cell types, their applicability as comparable universal indicators of cellular senescence across immune cell subsets remains uncertain as we continue to uncover the role these markers play in immune cell processes. Significant upregulation of DNA damage response pathways and epigenetic modifications, however, have demonstrated to be strong indicators of senescence without inherent bias towards specific cell types or conditions (Mah et al., 2010; Parris et al., 2015; Paluvai et al., 2020). Additionally, the accumulation of DNA damage and epigenetic modifications are considered as two of the main drivers of cellular senescence in immune cell subsets during aging (Aird and Zhang, 2013; Paluvai et al., 2020; Barbu et al., 2021; Protsenko et al., 2021).

Considering this, our study aimed to evaluate markers indicating proliferative arrest, dsbDNA damage foci, and SAHF at the single-cell level using imaging-based flow cytometry to achieve a more fine-grained analysis of iSC phenotypes, as described earlier. Given that MDD has been associated with a plethora of aging and cellular senescence hallmarks (Diniz et al., 2018; Diniz et al., 2019; Gonçalves et al., 2021; Mendes-Silva et al., 2021; Vieira et al., 2021; Diniz et al., 2022; Lorenzo et al., 2023; Seitz-Holland et al., 2023), we hypothesized that individuals with MDD would have a greater proportion of iSCs within the total PBMC compartment when compared to age-matched controls. Interestingly, this overall proportion of total PBMC iSCs between MDD and control groups was not different (3.24% compared to 2.42%, respectively). When characterized by iSC phenotypes, contingent on levels of dsbDNA and SAHF within each non-proliferating cell, however, we noticed significant differences in the distribution of iSC Hi, Int, and Lo populations between MDD and controls. We found that older adults with MDD had a higher prevalence of cells with iSC Hi senescence markers, in particular, in the proportion and number of monocytes and number of CD4 T cells. They also had a reduction in the proportion and number of iSC Int CD8 T cells.

There is limited understanding regarding declines in innate and adaptive immune cell responsiveness and function in MDD, even less so in older adults, especially beyond the scope of inflammation. Evidence from a 2018 study suggests, however, that there is a decrease in overall CD4 T-cell receptor repertoire diversity but not in CD8 T cells, effectively constraining their ability to respond to pathogens with MDD (Patas et al., 2018). Another study reported a significant reduction in respiratory and glycolytic capacity in CD3^+^ cells in patients with MDD (Gamradt et al., 2021). Since T-cell subsets are dependent on various metabolic programs to differentiate and mediate proper effector or helper functions, this could potentially push T-cells to have a stronger regulatory profile, ultimately diminishing essential effector functions to fight infection in depression. A 2013 study found that monocytes from depressed individuals were significantly less reactive to lipopolysaccharide *in vitro* when compared to those from healthy control patients (Lisi et al., 2013). Although focused on phenotypic characteristics, the results from this current study could also suggest the potential for skewed immune cell responses that warrant deeper investigation.

Although further studies are needed to evaluate the functional implications of phenotypic changes observed for each cell subset included in this study, it is possible that overall, iSC Hi cells may lack the ability to re-enter the cell cycle attributed to extensive DNA damage and SAHF indicative of an irreversible commitment to senescence. iSC Int cells also exhibit discernable levels of dsbDNA foci and SAHF, although to a lesser degree than iSC Hi cells, suggesting a transitional state where cells may either progress towards senescence due to accumulating DNA damage or retain the potential for repair and cell cycle re-entry under favorable conditions. iSC Lo cells display minimal to negligible levels of dsbDNA and SAHF, likely making them not senescent with the ability for DNA damage repair and cell cycle re-entry or positioning them at the very early stages of senescence. Taken together, this could indicate that the relationship between MDD pathophysiology and cellular senescence relies not necessarily in quantity of cells expressing senescence markers, but in subtype and degree of stress and damage experienced to induce and reinforce a senescence phenotype.

Based on our findings, we propose that individuals with MDD may experience a phenotypic shift from immune cells with intermediate to high senescence levels in contrast to non-depressed controls. It is also possible that cellular stress from persistent inflammation and other factors associated with MDD may induce greater DNA damage, driving immune cells to commit to a senescence phenotype or increasing their susceptibility to senescence. There is also a potential that the persistent stress associated with MDD can lead to impairment in the effective clearance of iSC Hi cells, whereas they are cleared in control individuals. Overall, our findings add to the growing literature supporting the relationship between cellular senescence characteristics and MDD (Diniz et al., 2019; Diniz et al., 2022; Lorenzo et al., 2023; Seitz-Holland et al., 2023). We expand these previous findings by showing evidence of elevated cellular senescence characteristics within total circulating immune cells and CD4, CD8, and monocyte subsets based on analysis at single-cell resolution.

While acknowledging the caveat that mRNA expression may not directly reflect protein expression, we discerned distinct association patterns between inflammatory cytokine mRNA expression and various iSC phenotypes. In general, we found a robust positive correlation between several inflammatory cytokines (e.g., *IL-5*, *IL-8*, and *CCL-2*) and iSC Hi CD4 T cells and monocytes. In contrast, there was a strong negative correlation between these cytokines and iSC Int and Lo CD4 T cells, CD8 T cells, and monocytes. These correlations were notably accentuated among individuals with MDD in contrast to non-depressed controls. Our findings suggest that MDD in older adults is associated with increased iSC characteristics in different immune cell subsets, accompanied by dysregulation of immunoinflammatory control at the mRNA level within these subsets. Finally, our findings support the notion that an “aged” and dysregulated immune system has the potential to significantly contribute to the elevated pro-inflammatory state associated with MDD (Diniz et al., 2016), the neuroprogressive nature of this disorder, and its long-term adverse outcomes (Morris et al., 2019; Lambert et al., 2022).

Our study results should be viewed in light of its limitations and opportunities for further investigation. First, we included a small sample size that may limit the generalization of our findings. Our analyses were also performed on unstimulated cells to capture differences as close to basal level as possible, although stimulating immune cells may have generated more striking results. In this preliminary investigation, we did not include functional measures of iSC cells such as response to stimulus or foreign antigens *in vitro*. As each cell type maintains its own responsive and immunologically protective capacity, this is a very important future step for characterizing immune cells in MDD and other conditions. To this end, it would also be interesting to determine if the differences in proportion of iSC phenotypes were also observed across naïve and memory subsets of T-cells between MDD and control groups.

Conducting functional analyses to ensure cells could not re-enter the cell cycle would also be important to explore, particularly given the diverse iSC phenotypes we describe. As discussed earlier, each iSC phenotype may possess a potential for cell-cycle re-entry, potentially challenging their characterization as true senescent cells. We focused on cellular senescence markers related to DNA damage (i.e., lack of proliferation, SAHF, and dsbDNA) and did not include other commonly used markers such as telomere length, SA-β-gal, p16^INK4a^, or p21^Cip1^. While our rationale still stands to include reliable reported markers of DNA damage foci and SAHF, it would be ideal to incorporate as many markers of cellular senescence as possible to confirm the senescence phenotype. Lastly, given the exploratory nature of some analyses, especially the correlation analyses, we did not correct the *p*-value for multiple comparisons. However, the significant associations were all moderate to strong reducing the risk of type I statistical error.

In conclusion, we found a higher prevalence of monocytes, CD4, and CD8 T cells with high expression of senescence markers in MDD and a shift from intermediate to high iSC phenotype in this population. We also found that PBMCs in older adults with MDD have higher mRNA expression levels of key inflammatory mediators, which could contribute to disease and pathogenesis associated with MDD. Our findings add to growing evidence showing significant abnormalities in multiple hallmarks of biological aging in MDD. Finally, the altered senescence profile observed in CD4 T cells, CD8 T cells, and monocytes of older adults with MDD suggests that targeting cellular senescence pathways in immune cells could be a potential avenue for therapeutic intervention in MDD.

## Data Availability

The raw data supporting the conclusion of this article will be made available by the authors, without undue reservation.
