# Supplementary figures and images for: Unraveling the association between major depressive disorder and senescent biomarkers in immune cells of older adults: a single-cell phenotypic analysis
Source: Front Aging. 2024 Apr 11;5:1376086. doi: 10.3389/fragi.2024.1376086 (PMC11043554; doi:10.3389/fragi.2024.1376086)

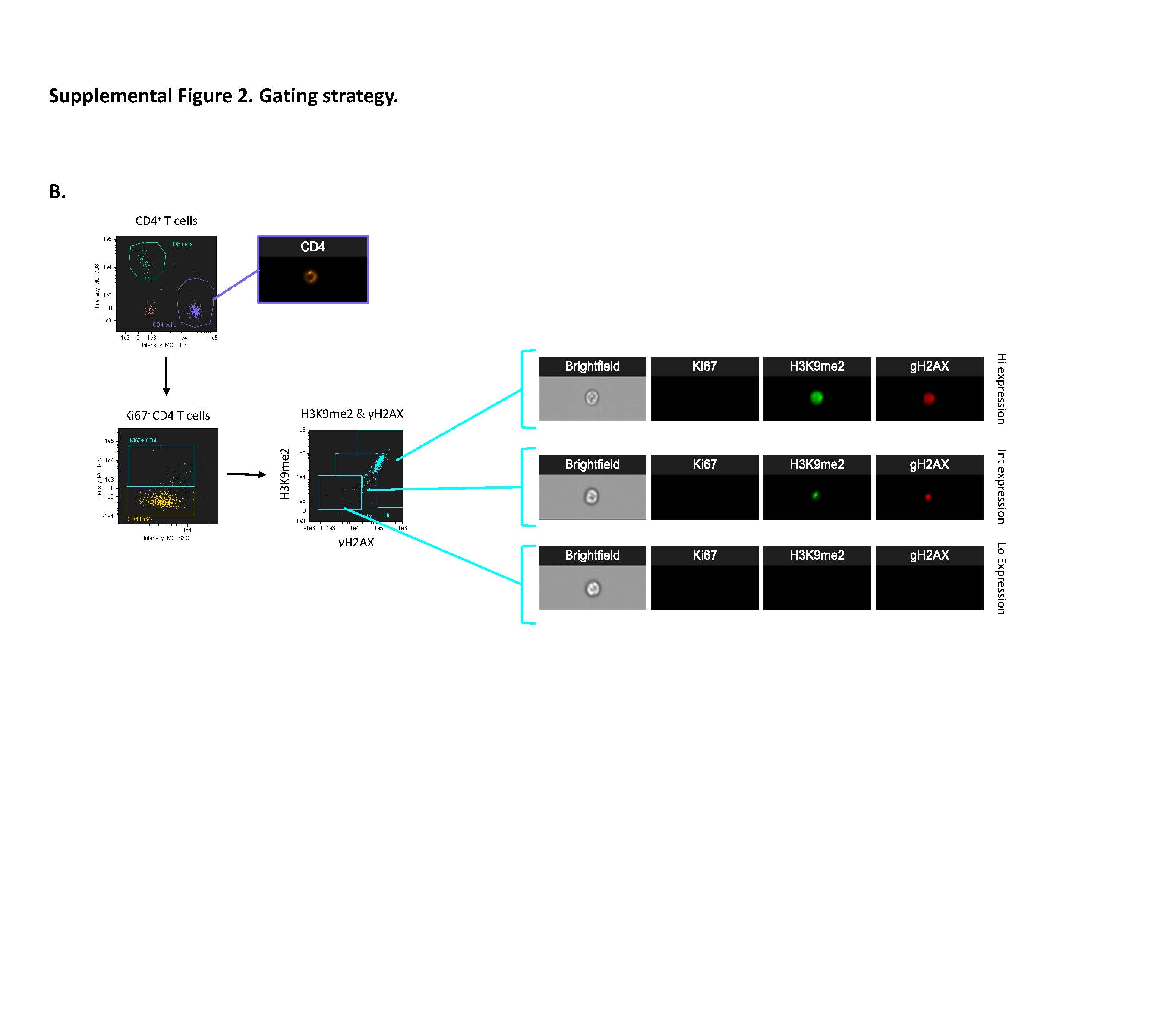

Supplement: Supplementary file 1 [file Image3.jpg]

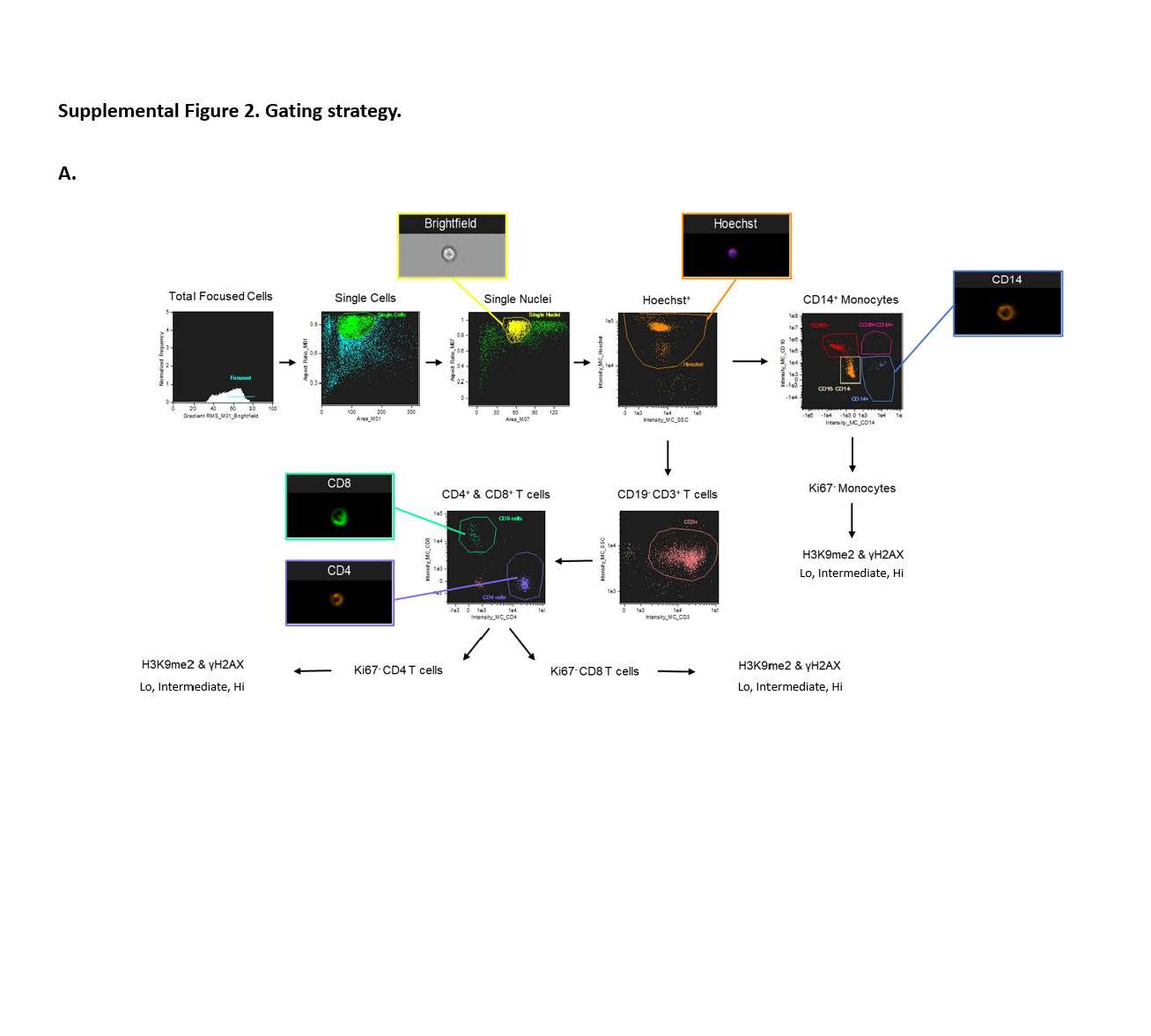

Supplement: Supplementary file 2 [file Image2.jpg]

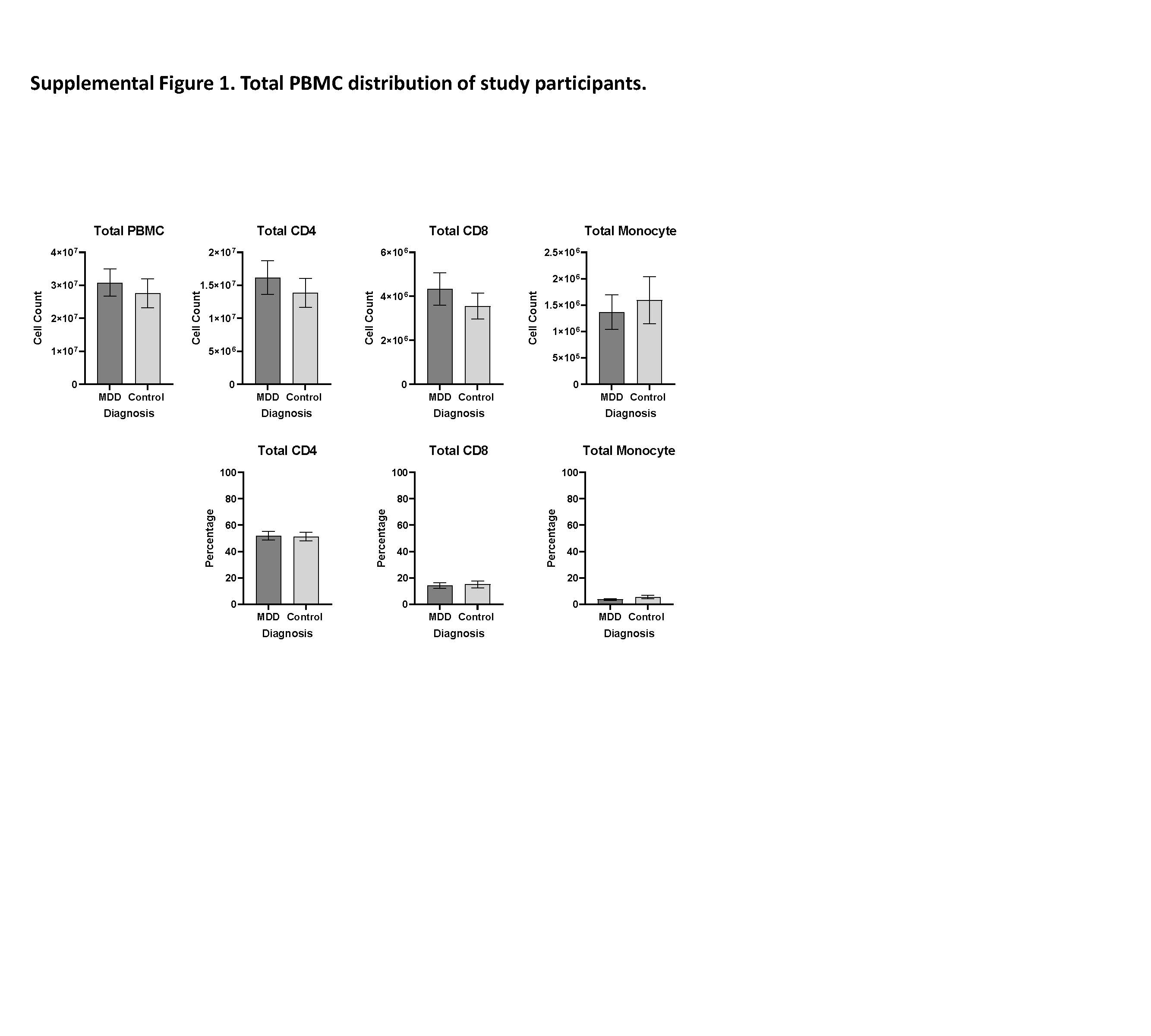

Supplement: Supplementary file 3 [file Image1.jpg]
